# Electronic nicotine delivery system (ENDS) liquid nicotine exposure in young children presenting to US emergency departments, 2018

**DOI:** 10.1186/s40621-019-0219-6

**Published:** 2019-10-21

**Authors:** Joanne T. Chang, Brian L. Rostron

**Affiliations:** Center for Tobacco Products, Office of Science, Document Control Center, US Food and Drug Administration, Building 71, Room G335, 10903 New Hampshire Avenue, Silver Spring, MD 20993-0002 USA

**Keywords:** ENDS, E-cigarettes, NEISS, CPSC, E-liquid, FDA, Liquid nicotine poisoning

## Abstract

**Background:**

Increased use of electronic nicotine delivery systems (ENDS) in the United States (U.S.) has been related to acute adverse events from liquid nicotine exposure. Previous studies have reported on these events through 2017.

**Findings:**

We used 2018 National Emergency Injury Surveillance System data to generate national estimates with 95% confidence intervals (CIs) of ENDS liquid nicotine-related poisonings among children under age 5 treated in U.S. hospital emergency departments (EDs). In 2018, an estimated 885 (95% CI: 397–1374) poisoning cases related to liquid nicotine among children under 5 were treated in U.S. EDs, which was a non-statistically signficant increase from 2017 (411 poisoning cases, 95% CI: 84–738). The most common route of exposure was through ingestion (99.4%). The majority of cases were treated and released from the hospital (90.0%), 8.9% of the cases left the hospital without being seen, and 1.1% of the cases were treated and admitted to the hospital.

**Conclusions:**

This study provides updated national estimates of poisoning events related to liquid nicotine exposure that occurred in 2018 among children under age 5. Updated information from this study may complement public education efforts and prevent liquid nicotine exposure among children.

## Background

Electronic cigarettes (e-cigarettes) are battery-operated electronic nicotine delivery systems (ENDS) that use “e-liquid” that may contain nicotine and other additives that are heated and inhaled by users in aerosol form (US Food and Drug Administration n.d.-a). In 2018, an estimated 3.05 million high school students (20.8%) and 570,000 middle school students (4.9%) in the United States (U.S.) reported using ENDS in the past 30 days (Cullen et al. 2018); in 2017, an estimated 6.9 million U.S. adults (2.8%) were current ENDS users (Wang et al. 2018).

The increased use of ENDS has generated discussions about their potential risks and benefits for population health. The unintentional exposure of liquid nicotine among children under 5 could lead to potential harm to neurological development, cause symptoms that require medical care, and result in death (Eggleston et al. 2016; Morley et al. 2017). Our previous study (Chang et al. 2019) using National Electronic Injury Surveillance System (NEISS) data estimated that 4745 liquid nicotine poisoning cases in children under age 5 presented to U.S. hospital emergency departments (EDs) from 2013 to 2017. Additionally, other studies have used data from the National Poison Data System (NPDS) to evaluate ENDS poisoning in young children (Chatham-Stephens et al. 2016; Govindarajan et al. 2018; Kamboj et al. 2016; Wang and Rostron 2017); however, these studies were unable to calculate population estimates of ENDS liquid nicotine poisoning because NPDS is not nationally representative.

In this study, we use NEISS data from 2018 to update national estimates of U.S. hospital ED visits for poisoning events related to ENDS liquid nicotine in children under age 5.

## Methods

### Data source

Detailed information on NEISS methods has been published elsewhere (US Consumer Product Safety Commission 2017). In brief, NEISS, maintained by the U.S. Consumer Product Safety Commission (CPSC), collects nonfatal injury data from about 100 U.S. hospitals with at least six beds that are selected as a nationally-representative probability sample of approximately 5000 U.S. hospital EDs (Schroeder and Adlt 2001). Each case in the NEISS database includes information on demographics, diagnosis, disposition, affected body part, product code, and a 142-character text narrative of the incident.

### Case extraction

We have previously published a description of the approach used here (Chang et al. 2019). In brief, to identify ENDS liquid nicotine-related events, we conducted a text search of the NEISS data using the text narrative field, including the following keywords: “cig,” “e-cig,” “ENDS,” “e-cigarettes,” “vapor,” “vape,” “e-hookah,” “hookah-pen,” “e-pipe,” “juul,” “jew,” “pods,” “liquid,” “nicotine,” “juice,” “e-liquids,” and “eliquid.” To identify poisoning-specific events, diagnosis code 68 (“poisoning”) was used. Since the diagnosis code is not specific to liquid nicotine poisoning events, we reviewed the text narrative involving liquid nicotine cases and identified routes of exposure (ingestion, dermal, or ocular). We identified cases related to ingestion using keywords such as “drink,” “drank,” “ingest,” “chew,” “sip,” “swallow,” “suck,” “mouth,” and “tongue”; cases related to dermal exposure using keywords such as “skin” and “dermal”; and cases related to ocular exposure using keywords such as “eye” and “ocular.” All cases were identified using the “FIND” procedure in the SAS program (version 9.4), and reviewers manually checked these cases were coded correctly.

In addition, two reviewers independently checked all extracted events and excluded events related to cigarette poisoning, or events not related to liquid nicotine exposure (Chang et al. 2019; Corey et al. 2018).

### Statistical analyses

We calculated the weighted population estimates of poisoning using the PROC SURVEY procedure in SAS program, accounting for the sample weights and complex design. We then estimated the weighted distribution of these events by the following characteristics: age groups (younger than age 2 and ages 2–4), sex (male and female), race (White and other/not stated), disposition (treated and admitted, treated and released, and left without being seen), location (home, other locations, unspecified), and route of exposure (ingestion and other/not stated).

CPSC considers the estimates to be unstable if (US Food and Drug Administration n.d.-a) the estimate is less than 1200 (Cullen et al. 2018); the number of records used is less than 20; or (Wang et al. 2018) the coefficient of variation is greater than 33% (Schroeder and Adlt 2001).

## Results

In 2018, we found 26 liquid nicotine poisoning cases in the NEISS database, representing an estimate of 885 (95% CI: 397–1374) cases among children under 5 who were treated in U.S. hospital EDs (Table 1). Most injuries occurred among females (69.9%), Whites (62.3%), and those under age 2 (59.4%). The most common route of exposure was through ingestion (99.4%). The majority of cases were treated and released from the hospital (90.0%), 8.9% of the cases left the hospital without being seen, and 1.1% of the cases were treated and admitted to the hospital.

**Table 1:** Electronic nicotine delivery system (ENDS) liquid nicotine-related injuries treated in emergency departments in the United States in 2018 in children under age 5

Characteristics	Unweighted N	N (%)
All	26	885 (95% CI: 397–1374)^a^
Age		
Younger than age 2 years	17	526 (59.4)
Ages 2–4 years	9	359 (40.6)
Sex		
Male	15	267 (30.1)
Female	11	618 (69.9)
Race		
White	15	552 (62.3)
Other/not stated	11	333 (37.7)
Disposition		
Treated and admitted to a hospital	2	10 (1.1)
Treated and released	23	797 (90.0)
Left without being seen	1	78 (8.9)
Location		
Home	21	847 (95.7)
Not stated	5	38 (4.3)
Route of exposure		
Ingestion	25	880 (99.4)
Other/not stated	1	5 (0.56)

^a^CPSC considers the estimates to be unstable if (1) the estimate is less than 1200; (2) the number of records used is less than 20; or (3) the coefficient of variation is greater than 33%

After examining the case narratives, we found that five out of 26 unweighted cases had information on symptoms, where 3 cases had vomiting, and 2 cases had emesis, and five cases had information on nicotine dose or concentration, where 2 cases ingested 60 mL, 1 case ingested 10 mL, and 2 cases ingested 0.6 mg and 3 cotton filters, respectively. We found 21 cases reported the poisonings occurred at home.

## Discussion

We used nationally representative data to update the estimate of ENDS liquid nicotine exposure among children under 5 and we found about 900 weighted cases occurred in 2018. From 2017 to 2018, we observed that liquid nicotine poisoning cases increased from 411 (Chang et al. 2019) to 885. Previously, we documented a large drop in nicotine exposure cases from 2015 to 2017, coinciding with the implementation of the Child Nicotine Poisoning Prevention Act (CNPPA) (Frey and Tilburg 2016). Although this increase is non-statistically significant, this raises potential concerns regarding the effectiveness of existing regulations as well as public health education efforts to protect young children from accident liquid nicotine poisoning.

Our study has some limitations. First, our estimates do not represent cases not treated in EDs; thus, total cases are likely to be underestimated. Wang et al. (2019) (Wang et al. n.d.) have estimated that about 29% of ENDS-related adverse events in NPDS resulted in EDs, and two-third occurred among children under 5. Additionally, the weighted estimate is less than 1200, which CPSC considered a national estimate unstable and potentially unreliable. Second, because NEISS data do not include product codes specific to ENDS or injury symptoms, we had to use general keywords to capture these events, which may also underestimate the burden. Third, findings may not represent the rapidly changing ENDS marketplace. Fourth, three cases indicated “possible or unsure ingestion”; thus, it is unclear whether these children ingested liquid nicotine. Lastly, ENDS liquid nicotine poisoning cases can also occur in children older than age 5, but these cases were not captured in NEISS (US Consumer Product Safety Commission n.d.).

Despite these limitations, this study extends previous findings (Chang et al. 2019) by updating national estimates of the number of U.S. ED visits for poisonings related to liquid nicotine exposure among children under 5. Updated information from this study may complement public education efforts and prevent liquid nicotine exposure among children. The U.S. FDA asserted regulatory jurisdiction over all tobacco products including ENDS in 2016 (US Food and Drug Administration n.d.-b). As such FDA is actively investigating scientific issues that can inform regulatory activities related to ENDS. Continued surveillance of these short-term outcomes can inform regulatory and educational activities.

## Data Availability

We analyzed the 2018 NEISS public use data file available from CPSC’s website under “Archived Full NEISS data by year” https://www.cpsc.gov/Research%2D-Statistics (last accessed 31 May 2019).

## Abbreviations

CDC: Centers for Disease Control and Prevention
CI: Confidence Interval
ED: Emergency Department
ENDS: Electronic Nicotine Delivery System
NEISS: National Electronic Injury Surveillance System
NPDS: National Poison Data System
U.S. CPSC: U.S. Consumer Product Safety Commission
U.S.: United States
